# Supplementary figures and images for: Modeling the impact of neuromorphological alterations in Down syndrome on fast neural oscillations
Source: PLoS Comput Biol. 2024 Jul 5;20(7):e1012259. doi: 10.1371/journal.pcbi.1012259 (PMC11253980; doi:10.1371/journal.pcbi.1012259)

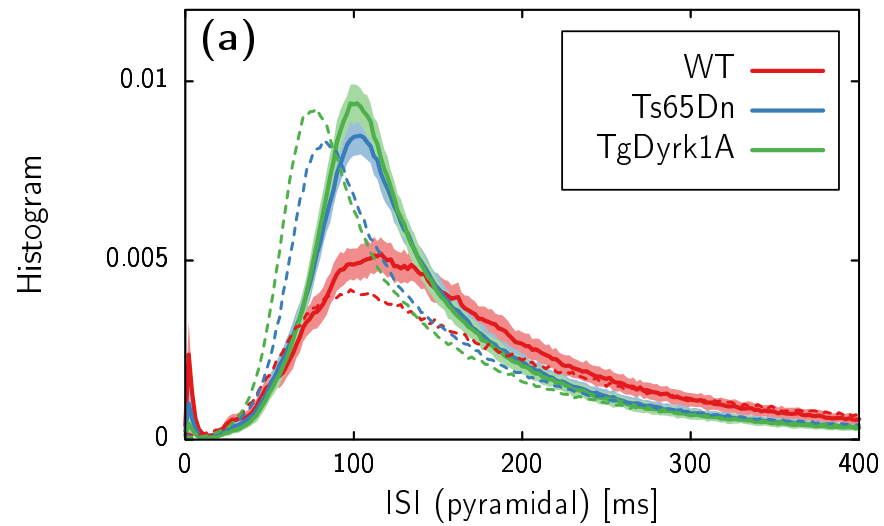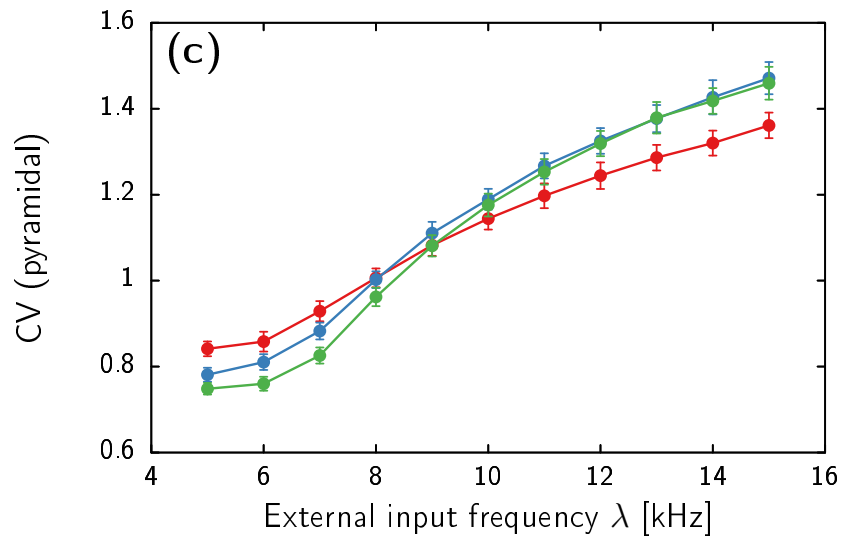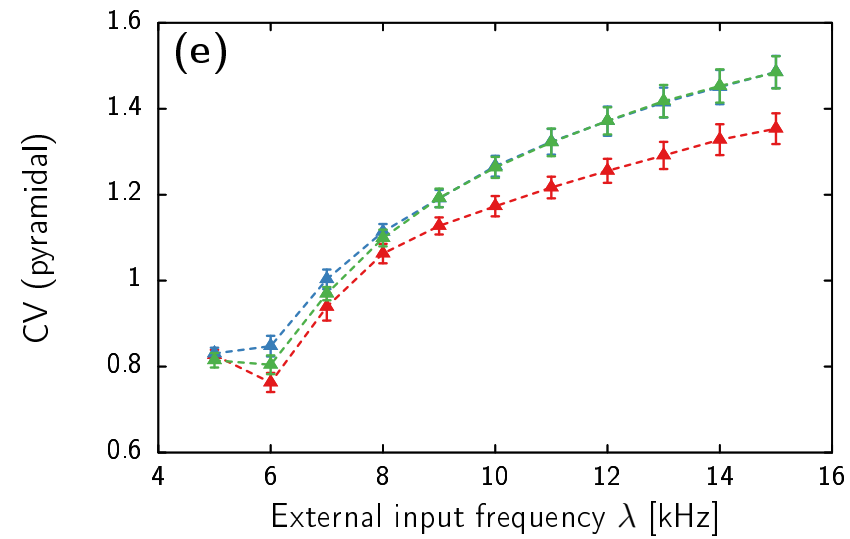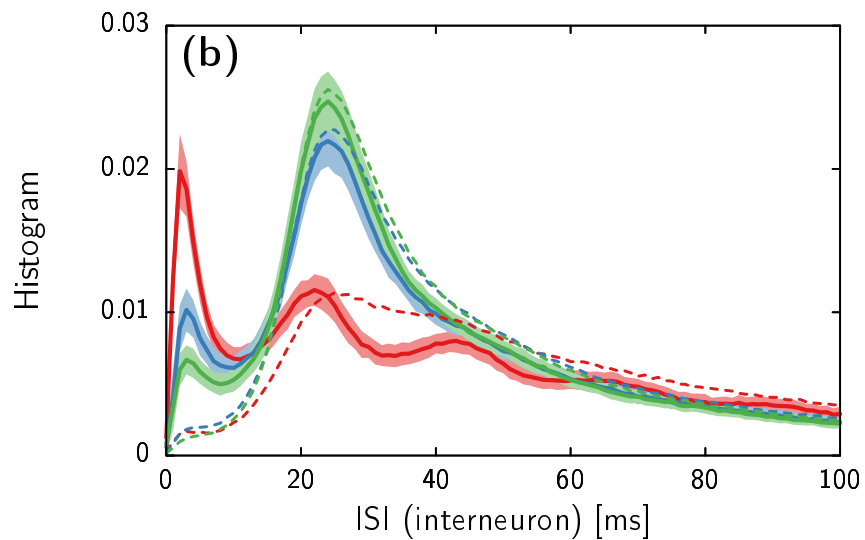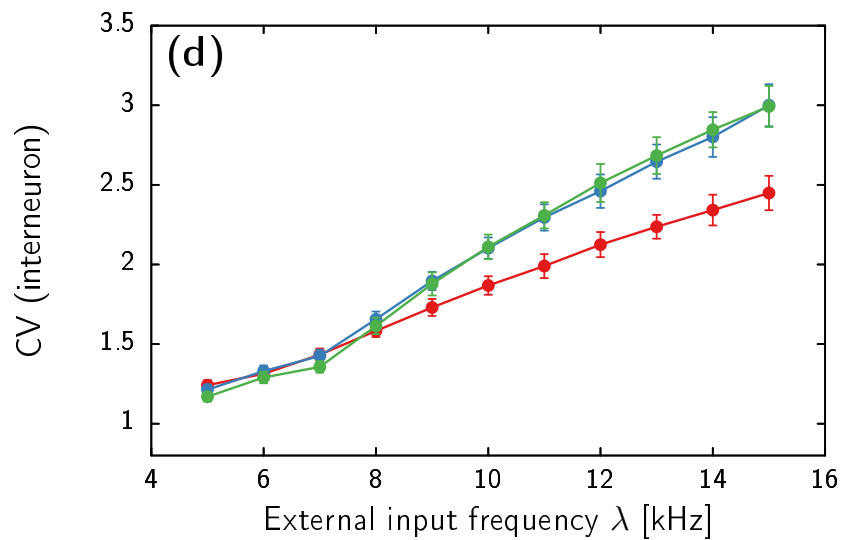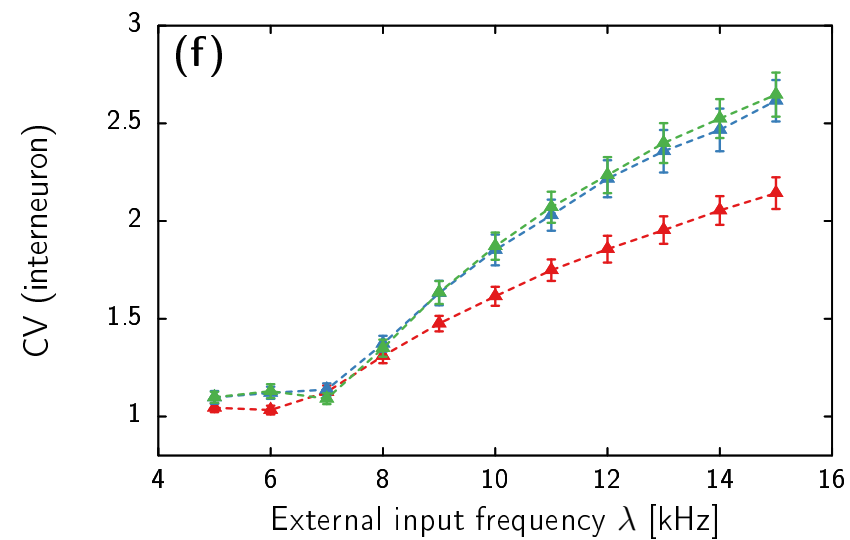

Supplement: S1 Fig — (a,b) Interspike interval (ISI) distribution corresponding to pyramidal neurons (a) and inhibitiory neurons (b) for λ = 9 kHz. Each curve corresponds to the average of 100 histograms corresponding to 10 independent realizations of the noise for 10 different topologies. Shaded regions indicate the standard deviation among the samples. Red, blue, and green correspond to the morphological parameters of the WT, Ts65Dn, and TgDyrk1A cases, respectively. Dashed lines correspond to simulations with recurrent inhibitory synapses reduced to 0.3 of the original value. (c-f) Coefficient of variation (CV) of the ISI distribution of pyramidal neurons (c) and inhibitory neurons (d) for different values of external input λ. Symbols correspond to the average CV of 100 ISI distributions, and errorbars indicate the respective standard deviation. Panels (c) and (d) correspond to the default network parameters, whereas panels (e) and (f) correspond to simulations with recurrent inhibition reduced to 0.3 of the original value. (PDF) [file pcbi.1012259.s001.pdf]

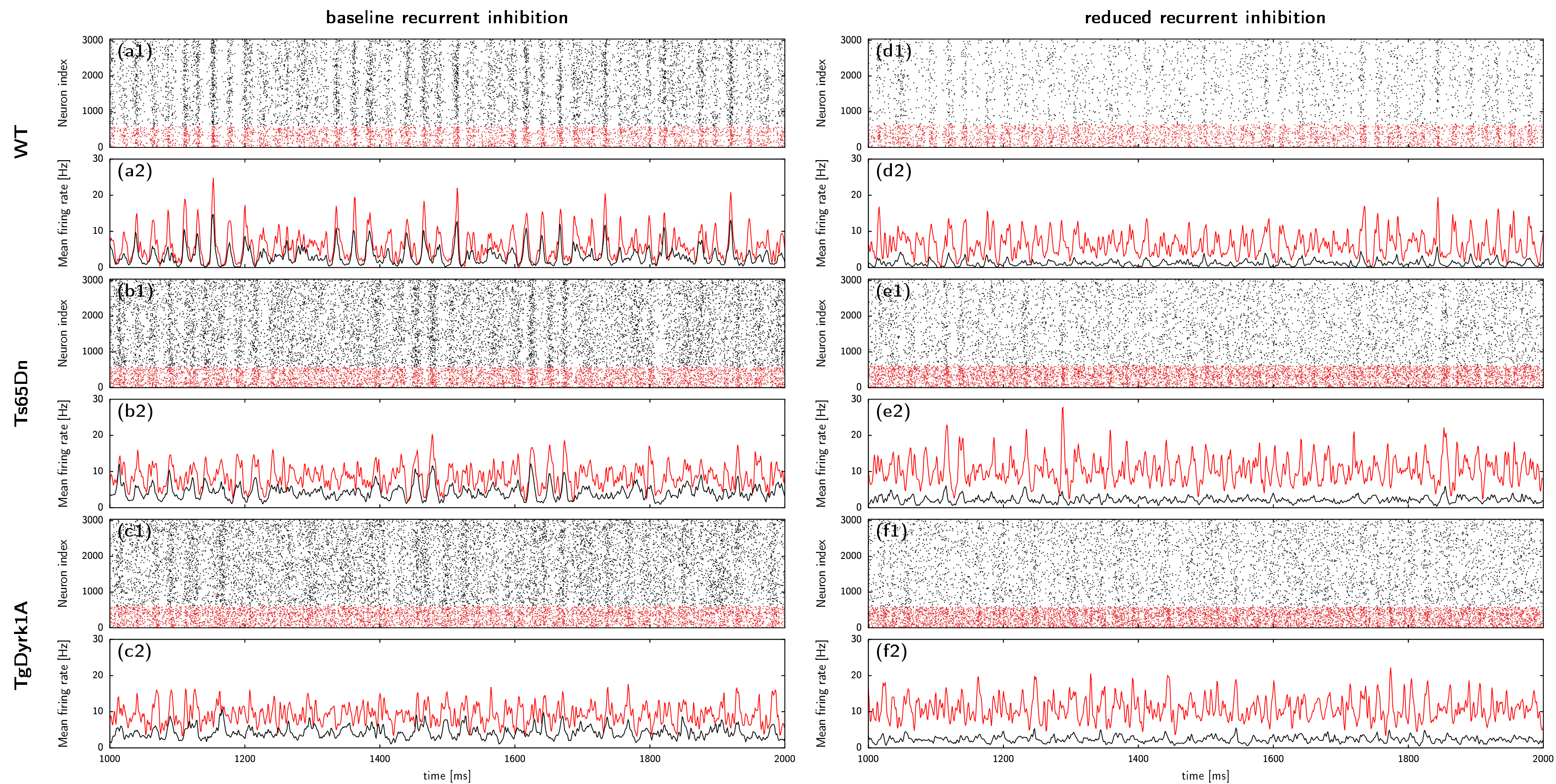

Supplement: S2 Fig — Raster plots and mean firing rate of the pyramidal (black) and inhibitory interneurons (red) for the WT (panels (a) and (d)), Ts65Dn (panels (b) and (e))), and TgDyrk1A (panels (c) and (f)) morphologies. Panels (a-c) correspond to simulations with unperturbed recurrent inhibition (same as in Fig 4(a)–4(f)), and panels (d-f) correspond to recurrent inhibitory synapses reduced to 0.3 of the original value. (PDF) [file pcbi.1012259.s002.pdf]

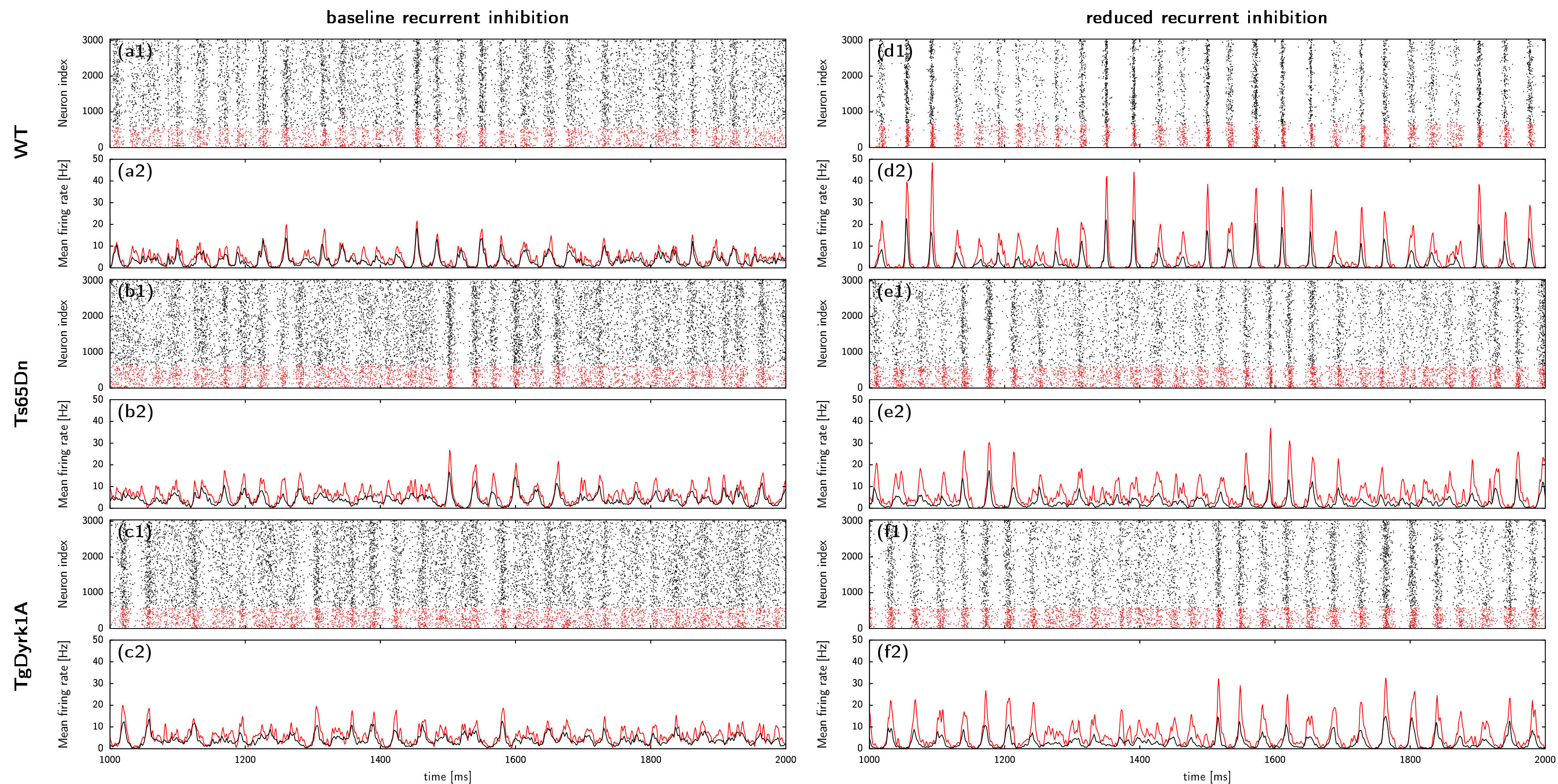

Supplement: S3 Fig — Raster plots and mean firing rate of the pyramidal (black) and inhibitory interneurons (red) for the WT (panels (a) and (d)), Ts65Dn (panels (b) and (e))), and TgDyrk1A (panels (c) and (f)) morphologies. Panels (a-c) correspond to simulations with unperturbed recurrent inhibition, and panels (d-f) correspond to recurrent inhibitory synapses reduced to 0.3 of the original value. (PDF) [file pcbi.1012259.s003.pdf]

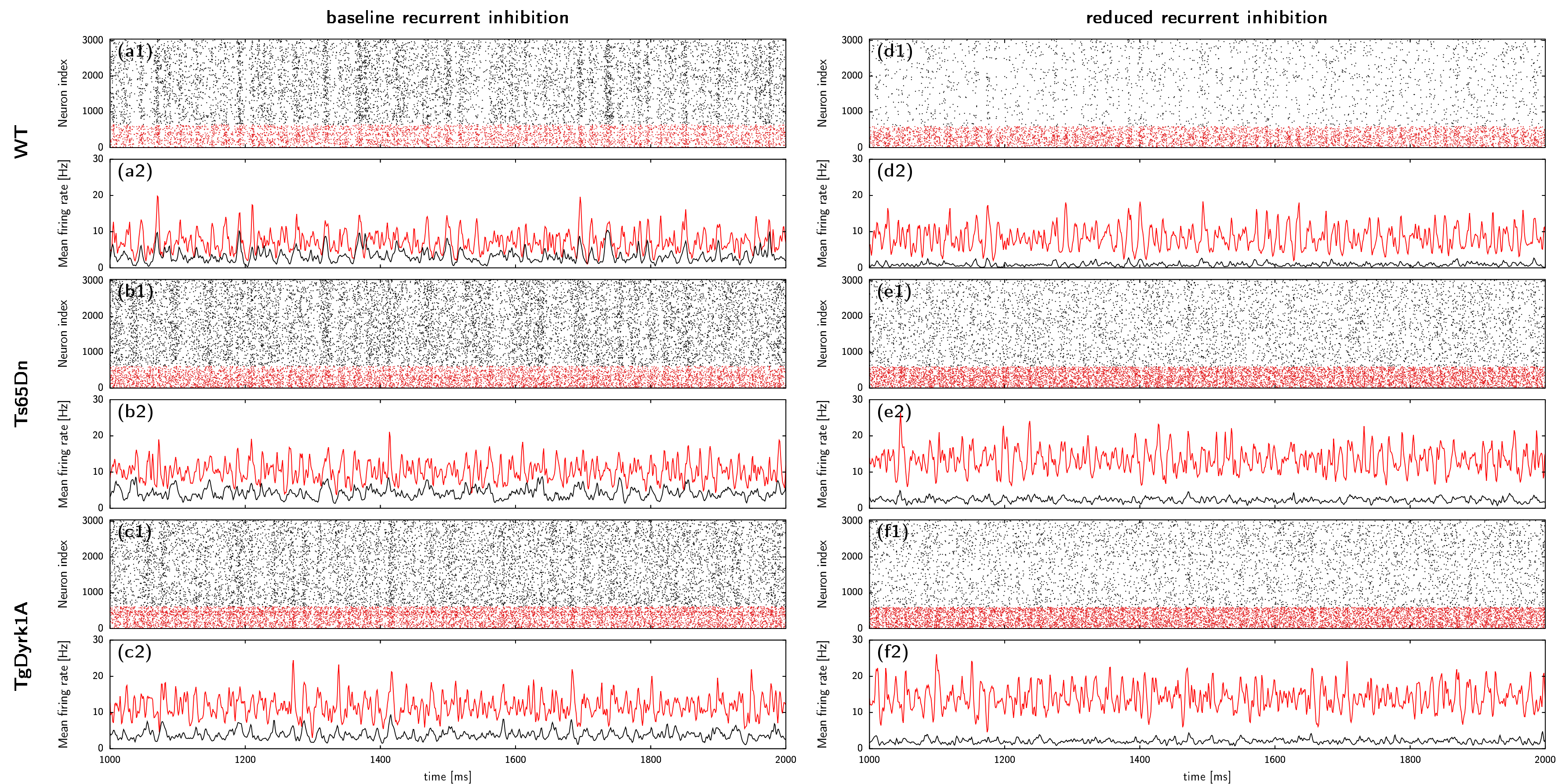

Supplement: S4 Fig — Raster plots and mean firing rate of the pyramidal (black) and inhibitory interneurons (red) for the WT (panels (a) and (d)), Ts65Dn (panels (b) and (e))), and TgDyrk1A (panels (c) and (f)) morphologies. Panels (a-c) correspond to simulations with unperturbed recurrent inhibition, and panels (d-f) correspond to recurrent inhibitory synapses reduced to 0.3 of the original value. (PDF) [file pcbi.1012259.s004.pdf]

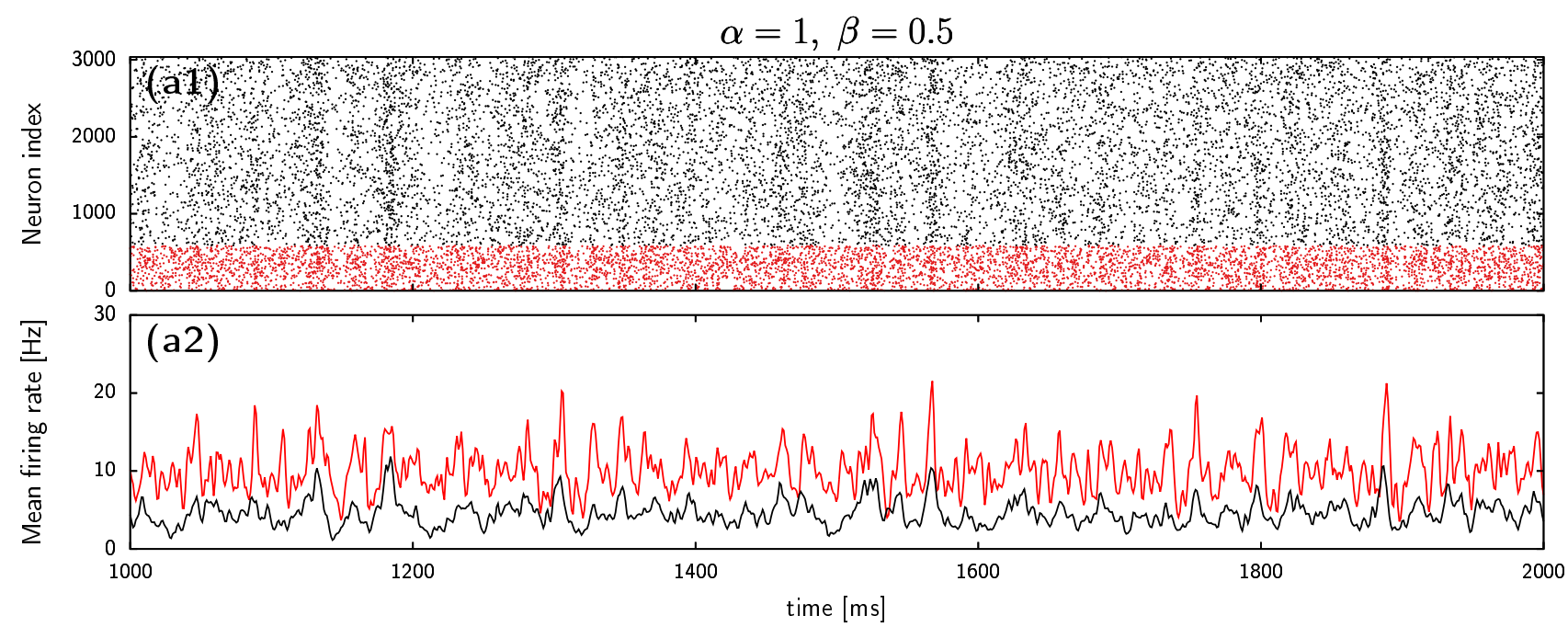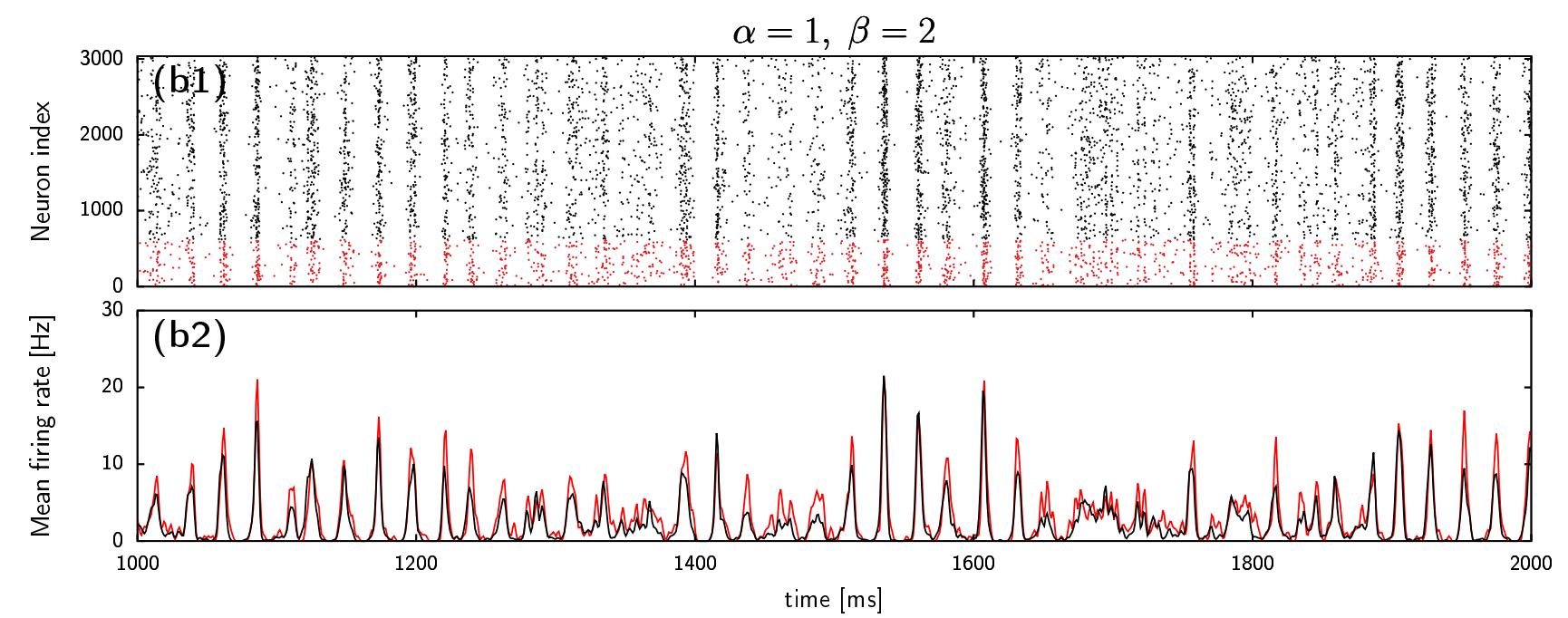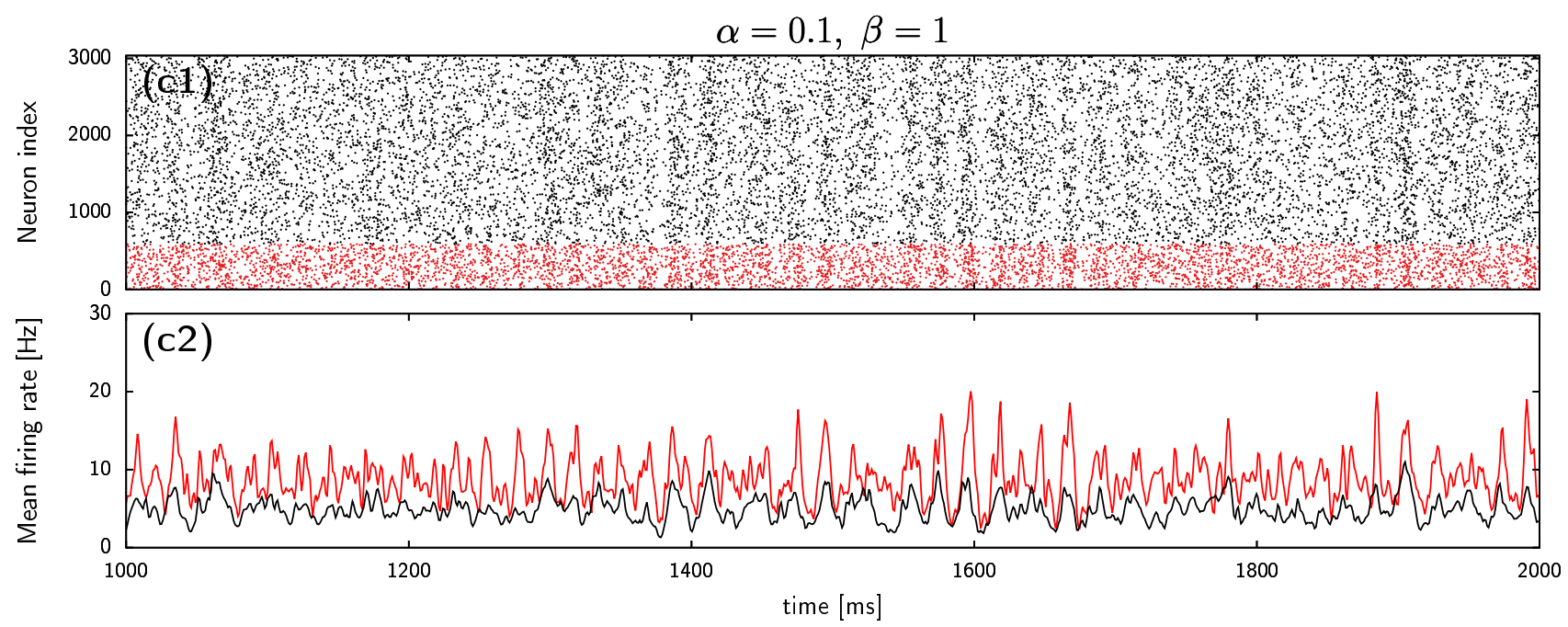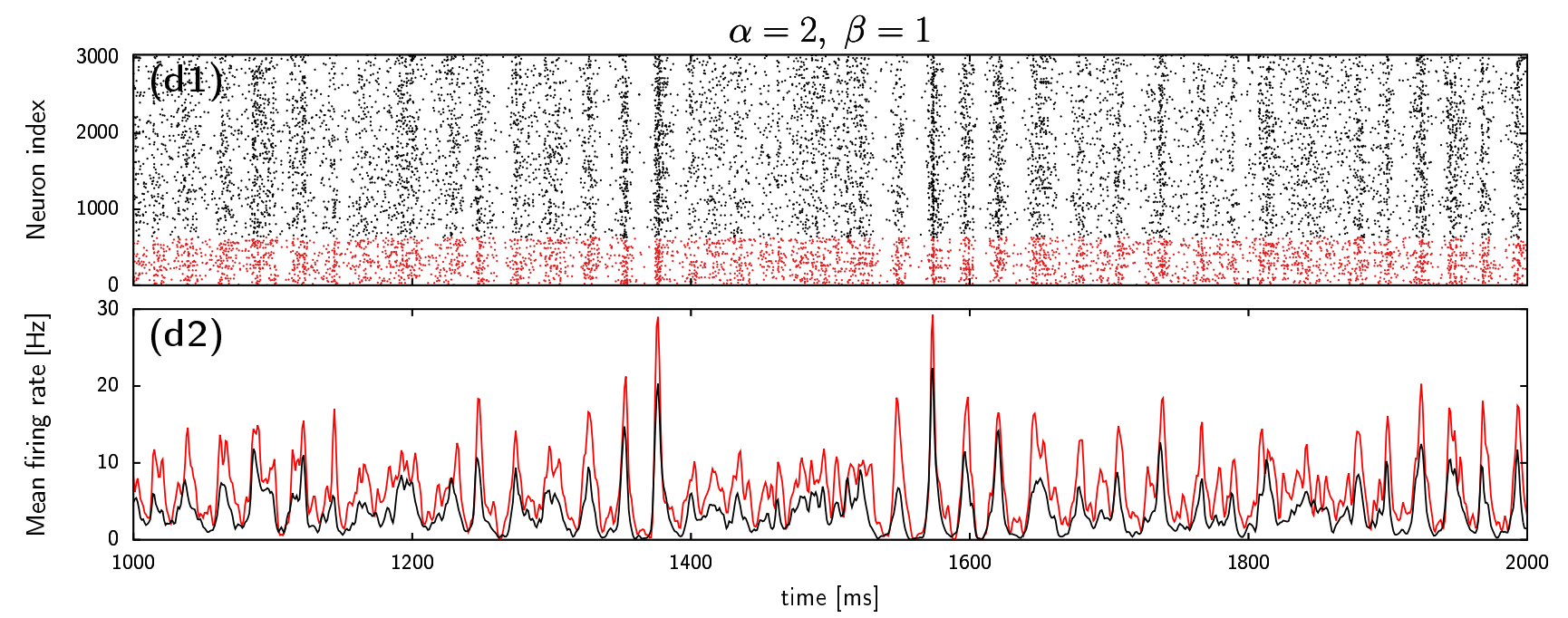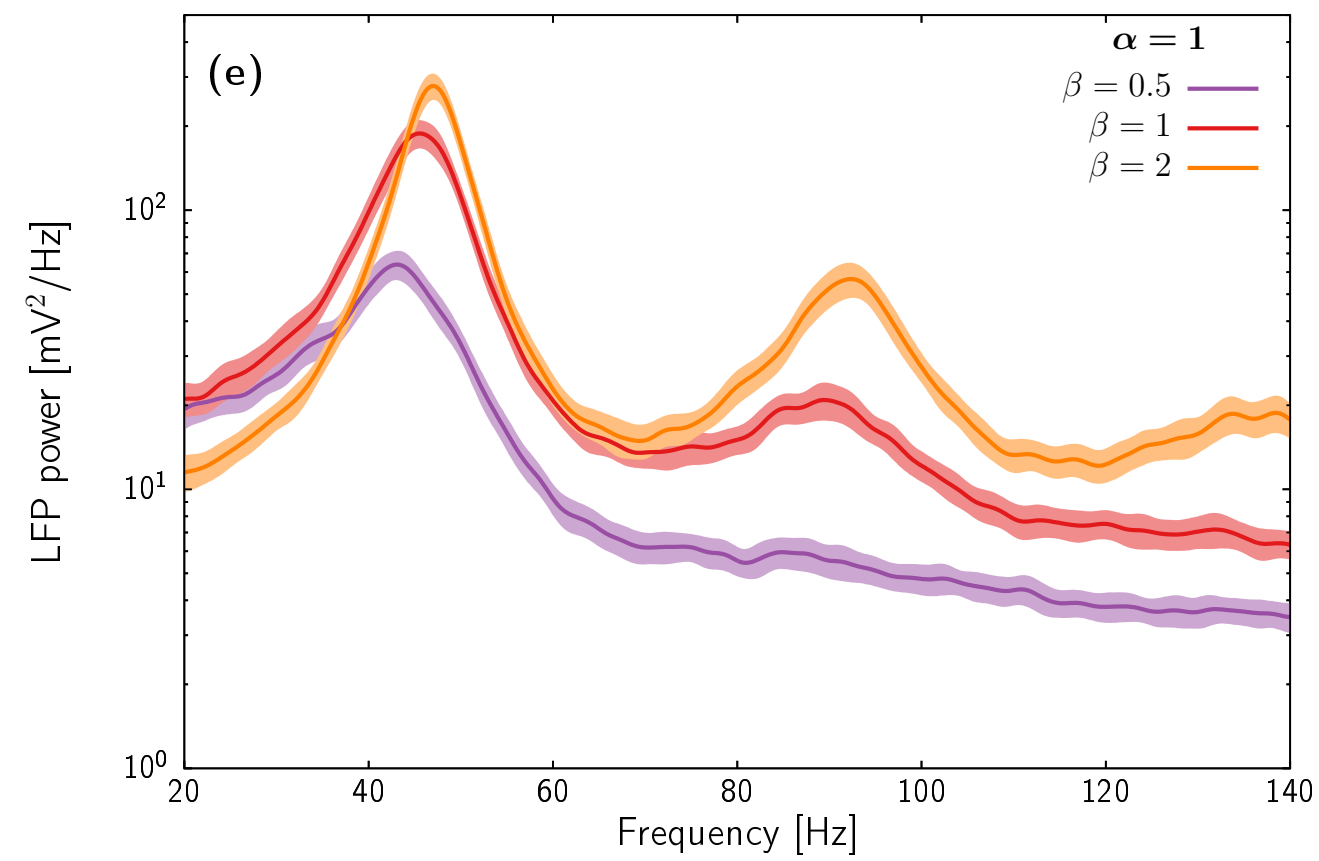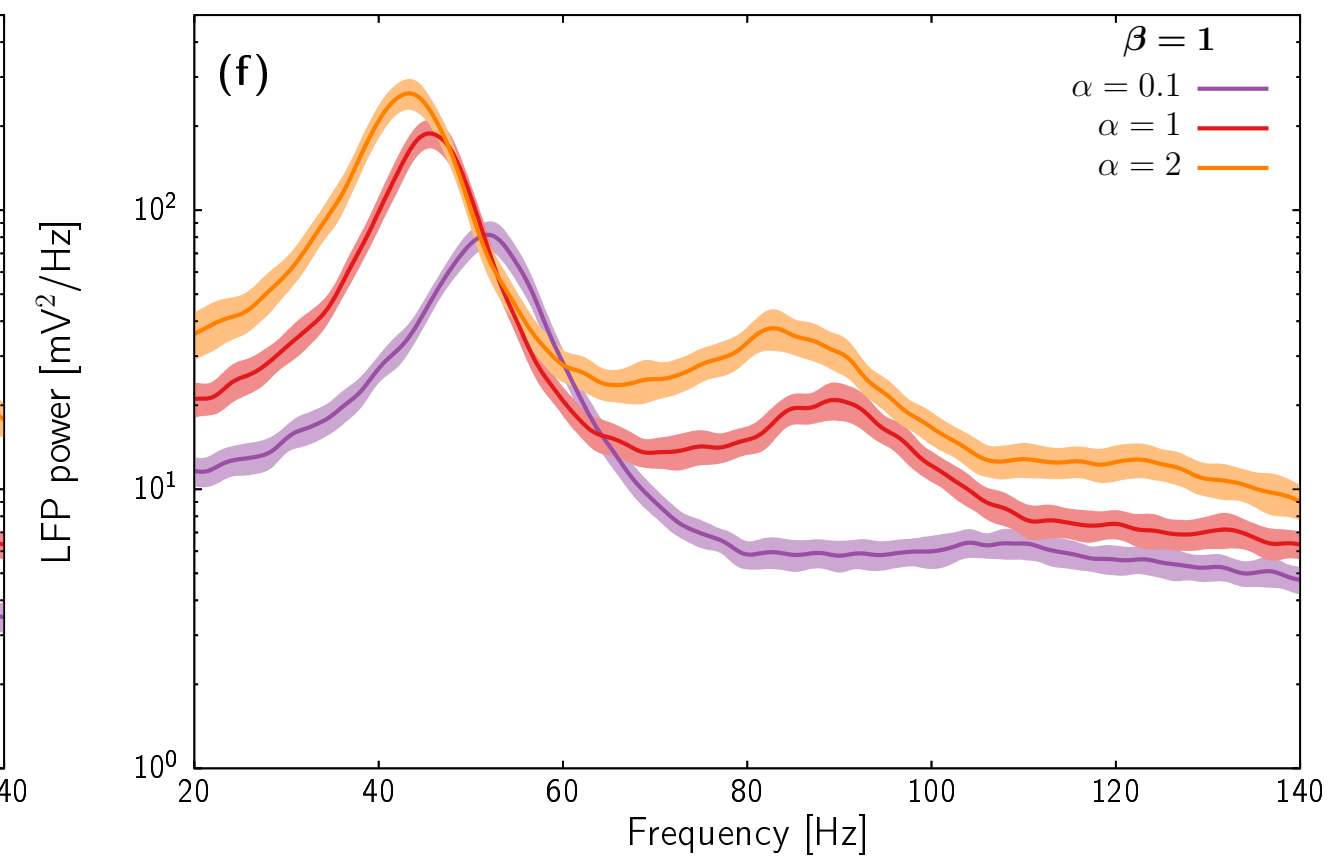

Supplement: S5 Fig — (a-d) Raster plots and mean firing rate of the pyramidal (black) and inhibitory interneurons (red) for network topologies generated with different values of the SCP scaling parameter α and the scaling of the mean dendritic tree size with respect to WT β. Rest of the parameters as in Fig 5. (e,f) Average power spectra of the LFP signal corresponding to Fig 5 for specific values of α and β. (PDF) [file pcbi.1012259.s005.pdf]
