# Supplementary material for: Modeling the impact of neuromorphological alterations in Down syndrome on fast neural oscillations
Source: PLoS Comput Biol. 2024 Jul 5;20(7):e1012259. doi: 10.1371/journal.pcbi.1012259 (PMC11253980; doi:10.1371/journal.pcbi.1012259)
Supplement: Attachment — Submitted filename: response_letter.pdf [file pcbi.1012259.s009.pdf]

Point-by-point response to PCOMPBIOL-D-24-00075: "Modeling the impact of neuromorphological alterations in Down syndrome on fast neural oscillations"

We appreciate all the great comments and constructive suggestions. We have addressed the raised concerns as detailed in the responses below, adding the needed supporting materials.

1. We modified Fig. 4 (previously Fig. 3) and added five supplementary figures to describe the network emergent dynamics.
2. We added a new section to report and discuss the topology changes between the three genotypes, including a new figure (Fig. 3).
3. We generated supplementary results assessing how the variation in the synaptic contact probability can affect the gamma oscillation for a fixed dendritic tree size.
4. We addressed other minor issues point by point.

Comments from Reviewer #1

Overall comment: In this manuscript the authors propose a computational model reproducing the dynamics of a cross-sectional layer of the mouse motor cortex. In particular they design the network connectivity such that it accounts both for the mean dendritic radius and the spine density of the single neurons, starting from the data of three mouse models: wild-type, transgenic and trisomic. The idea underlying the manuscript is to characterize the effects in the emergent dynamics of morphological alterations present in neurons in case of cognitive disorders, like Down syndrome (DS). In particular they analyse the emergence of gamma activity and how this activity diminishes in DS models. The findings support the notion that microscopic circuitry abnormalities contribute to the disruption of network dynamics in DS.

The manuscript is well written and interesting and I appreciate the attempt to fill a gap trying to understand the relationship between neromorphological alterations and impaired brain function.
However, I think the manuscript needs to be improved in order to be published, because a characterization of the dynamics emergent in the network (in terms of raster plots and time-behavior of macroscopic variables) is completely missing.
In the following there is a detailed list of my remarks and questions.

Overall Response: We deeply appreciate the positive comments regarding the contributions of this manuscript.

In the new version we have modified several parts of the Results section and have generated a set of supplementary figures in order to address the main comments raised. Please, see point-by-point responses below.

Comment 1.a: Since the authors propose a novel computational model, I think it is necessary to characterize the dynamics emergent in the simulated network in more detail. I understand that such characterization may distract the reader from the main message, but such analysis can be reported mainly in the Methods section or in some Supplementary figures, without impacting on the main results.

Response: We modified Fig 4 (former Fig 3) in order to illustrate the dynamics of the network in the main manuscript. Also, we added Supplementary Figures S1-5 showing further examples of the network dynamics and their spiking regularity. In particular, we added raster plots and the corresponding mean firing rate time series for excitatory and inhibitory neurons in the 3 mouse models. See detailed responses below.

Comment 1.b: Since the authors are using a network composed of almost 3000 neurons, where are present both regular spiking pyramidal neurons and fast spiking interneurons, I suggest to present separated results for the inhibitory and for the excitatory neurons. Please present a raster plot in time of both inhibitory and excitatory neurons for different parameters sets.
Are there some differences for the 3 mouse model cases?

Response: Thank you for this suggestion. The comparison of the raster plots between the 3 mouse model cases already indicates a decrease in synchronicity for the pathological cases in Fig 4(a-f). This can also be observed in Supplementary Figures S2-S4, which illustrate these changes upon modifying the external input and the recurrent inhibitory synaptic strength. Nonetheless, the power spectral analysis is still required to properly compare the dynamics of the three genotypes.

These new results are now discussed mainly in the paragraphs starting at lines 160 and 169, as well as the new sentence in line 238 for the case of reduced recurrent inhibition. We hope that these additional figures help to illustrate the dynamics of the network and their dependence on external input and recurrent inhibition.

Comment 1.c: Which are the dynamical regimes emerging in this context? Is gamma activity linked to a periodic oscillation regime?

Response: In the context of excitatory vs. inhibitory activity dynamics, our simulations show that gamma oscillations in our model emerge as collective stochastic oscillations.

This dynamical regime is therefore analogous to that analyzed in (Brunel 2000 and Brunel and Wang 2003), in which networks of excitatory and inhibitory neurons are analyzed. Since our model is finite and stochastic, we can not strictly speak about purely periodic oscillations, as the collective rhythm is quite irregular (as is usually the case in neural recordings). Nonetheless, there is a clear oscillatory component as revealed by the power spectral analysis. We discuss the nature of this regime and the role of noise in the paragraph starting in line 160.

Comment 1.d: Do the dynamical regimes change when reducing the recurrent inhibitory synapses? or when changing alpha and beta parameters?

Response: This is indeed an interesting question. Since the model is stochastic and finite, it is not possible to unequivocally determine differences between dynamical regimes. This would be possible in deterministic systems or performing finite-size analysis, which goes out of the scope of this work.

Nonetheless, based on Figs. 4 and 5 of the manuscript, as well as the additional Supplementary Figures S1-5, we conclude that the main nature of the collective rhythm is not altered in the different simulations we performed. In all cases we observe collective (noisy) gamma oscillations paired with irregular single-neuron activity. However, there are clear differences regarding the prominence of the oscillatory component and the regularity of the synchronization, specially, of excitatory neurons. These differences can be observed by changes on the external input frequency (see Fig 4 and S2-4), reduction of the recurrent inhibitory synapses (same figures), and also morphology changes (see Figs 6 and S5).

Comment 1.e: Moreover, please report the LFP in time (or spectrograms of the LFP in time) for the inhibitory and excitatory populations and calculate the coefficient of variation (CV) of the network for different set of parameters.

Response: All raster plots included in Fig 4 and Supplementary Figures S2-5 are accompanied by the mean firing rate of excitatory and inhibitory neurons. Notice that in the manuscript we compute LFP as the network-wide mean firing rate (see Eq. (11) in Methods), but we separate between inhibitory and excitatory components in the reported time series in order to allow for a better characterization of the dynamics, as suggested by the Referee. As noted in the new sentence in line 238, these results help to characterize how reduced inhibition affects gamma activity.

Additionally, we include examples of the interspike interval distribution and the corresponding coefficient of variation in Supplementary Figure S1. These results are discussed in the new paragraph starting in line 185 as well the new sentence in line 214.

Comment 2: Which is the biological reason underlying the choice of external input frequency lambda around 10 kHz? What does the external current account for? Does it take into account the signals from other cortical layers? Please explain.

Response: The external input parameter λ accounts for all inputs received in the simulated neurons, thus it implicitly accounts for both other cortical layers and also any inputs projecting to layer 2/3. We clarified this in the revised manuscript (lines 462-463).

Regarding the choice of this quantity, as described in Figure 4(b) and (c), we tested a range of external input frequencies to explore their impact on gamma oscillations in the simulated network. Based on this exploration, we found values between 8-12 kHz to provide robust gamma oscillations. Values larger than 12 kHz show more variability in the pathological cases (see Fig 4), whereas values smaller than 8 kHz produce collective oscillations in a frequency range slightly below 40 Hz. We therefore chose 9 kHz in most of the exemplary simulations, although slight variations of this parameter do not affect the main results.

Comment 3: In the Methods section w_j is used to identify the recovery variable, while w_{i,j} are the entries of the connectivity matrix. I would suggest not to use the same letter for different variables as it is now.

Response: Thank you for pointing this out. We fixed this by replacing the letter used for the recovery variable to "u" (see Equation (9) in Methods). We also changed the subindices to "i" for consistency.

Comment 4: Could you please provide some more details on the connectivity matrix used in the model? Which is the average connectivity in the network? Do excitatory and inhibitory neurons have the same average connectivity?
How much does it vary among the different trials?

Response: We added an entire new subsection "Morphology changes impact network connectivity" in the Results to provide context information of the structural connectivity, with a new Figure 3 and a new Table 3 (starting in line 120). Specifically, we show the degree and connectivity weight distributions of the network. In particular, we see significant changes in connectivity and degree distribution between genotypes. Nonetheless, inhibitory and excitatory neurons are not distinguished in the topology

generation algorithm, and therefore, both cellular types share the same connectivity profiles (see new lines 442-446 in Methods).

Comments from Reviewer #2

Overall comment: Clusella et al report about the link between changes in morphology and the disruption of gamma oscillations in DS. For this they developed a cortical-layer structural model to mimic the synaptic connectivity of a neural network of cross section of layer 2/3 of the motor cortex. The model is made of point neurons, including pyramidal and fast-spiking interneurons, and based on the empirical data on dendritic complexity and spine density obtained from wild-type (WT), transgenic (TgDyrk1A), and trisomic mice (Ts65Dn). The authors demonstrated that their model based on morphological changes replicates the disruption of gamma activity observed in the models. The model is based on a unique synaptic contact probability and can be used to reflect the 3 genetic conditions explored here. According to the model, the authors proposed that the reduction in the dendritic tress size is the main factor contributing to the gamma abnormalities in DS while the reduction in synaptic contact probability may have a second role.

Overall Response: We thank the reviewer for the positive comments regarding the significance of the project and this manuscript. We are thankful for the constructive and insightful suggestions in the comments below. We tried our best to accommodate your suggestions in the revised version of the manuscript. Please note that line numbers are specified for the revised version with markup.

Comment 1: It would have been of interest to show if the variation in the synaptic contact probability can affect the gamma oscillation for a fixed dendritic tree size.

Response: This is an interesting case, which was previously only shown in Fig 4 (Fig 5 in the new version). We agree that in this figure it is difficult to visualize the impact of leaving a fixed dendritic tree size (parameter β) while changing the SCP (parameter α). In order to further outline this case we incorporated Supplementary Figure S5, which shows specific raster plots and time series for changes in β only (panels a and b), and changes in α only (panels c and d). We also display the PSD for these cases (panels e and f). All these simulations show that changes in only one of the two morphological parameters already modify the synchronicity of the gamma activity. We now remark this in lines 293-295. Nonetheless, it has to be noted that each parameter has a different impact on the frequency of the oscillation, as already noted in lines 280-281.

Comment 2: Wild-type individuals should be written as “wt” not “WT”.

Response:
We thank the Referee for this suggestion. Nonetheless, since we are using "WT" as an acronym, following the convention in PLoS CB articles (see, e.g.,

https://doi.org/10.1371/journal.pcbi.1003895) we maintain "WT" (uppercase) in the revised version.

Comment 3: The gene dual-specificity tyrosine phosphorylation regulated kinase 1a gene should be written according to the international nomenclature Dyrk1a not Dyrk1A.

Response: We corrected this in the revised manuscript (line 34).

Comment 4: The full name of the models used in the manuscript should be given and not only short internal labels like TgDyrk1A and Ts65Dn with reference for the original source.

Response: The full name of Ts65Dn is "Ts(17(16))65Dn," but also B6EiC3Sn.BLi a/A-Ts65Dn, or B6C3.B-Ts65Dn. We have used Ts(17(16))65Dn (see line 32), but if the reviewer prefers others please let us know. As for TgDyrk1A, we used the line generated by Dr. Cristina Fillat in which the sMT-Ia/Dyrk1A chimeric gene was used. The original name was TgDyrk1A.

In both cases we have included the original publication (see Refs. [18] and [19] in the new version, which are cited in lines 32 and 33 respectively).
